# Pulse oximetry, racial bias and statistical bias: further improvements of pulse oximetry are necessary

**DOI:** 10.1186/s13613-022-00992-z

**Published:** 2022-02-22

**Authors:** Marco Ferrari, Valentina Quaresima, Felix Scholkmann

**Affiliations:** 1 Department of Life, Health and Environmental Science, University of L’Aquila, 67100 L’Aquila, Italy; 2 Biomedical Optics Research Laboratory, Department of Neonatology, University Hospital Zurich, University of Zurich, Zurich, Switzerland

We read with great interest the review by Tobin and Jubran [1]. We strongly agree about the urgent need to correct racial bias due to the negative impact of dark skin pigmentation on the accuracy of pulse oximetry, and the necessity to introduce new international medical standards. In contrast to pulse oximetry, the influence of melanin absorption in the epidermal layer is already corrected in transcutaneous bilirubinometry, a widely used screening method, based on multi-wavelength optical spectroscopy, employed to diagnose of neonatal hyperbilirubinemia [2]. Unfortunately, pulse oximetry devices without melanin correction, and having additional technical limitations, are widely in use in many countries during the current COVID-19 pandemic [3, 4]. Therefore, there is an urgent need that manufacturers would replace conventional two-wavelengths finger pulse oximeters with multi-wavelength systems implemented for melanin absorption correction. High-cost multi-wavelength finger pulse oximeters, named pulse CO-oximeters, capable of estimating blood levels of carboxyhemoglobin and methemoglobin, are already commercially available. The significance of methemoglobinemia has been reported for COVID-19 patients [5]. The introduction of low-cost multi-wavelength finger pulse oximeters with melanin correction is urgently necessary. Furthermore, manufacturers of pulse oximeters should be forced to release the calibration algorithm and calibration data employed in their instruments so that the public and the medical community using these devices are aware of the possible bias introduced by calibration algorithm and data.

## Data Availability

Not applicable.
